# Digital Phenotyping of Pain Modulation and Associations Among Personality, Attachment, and Behavioral Signatures: Cross-Sectional Study

**DOI:** 10.2196/91540

**Published:** 2026-03-13

**Authors:** Chie Kishimoto, Hani M Bu-Omer, Aya Nakae

**Affiliations:** 1 Presence Media Research Group Hiroshi Ishiguro Laboratories, Deep Interaction Laboratory Group Advanced Telecommunications Research Institute International Seika-cho, Soraku-gun, Kyoto Japan; 2 Global Open Lab Strategy Office Deep Interaction Laboratory Group Advanced Telecommunications Research Institute International Seika-cho, Soraku-gun, Kyoto Japan

**Keywords:** pain modulation, personality, attachment, digital phenotyping, precision management, susceptibility

## Abstract

**Background:**

The transition from acute to chronic pain often reflects a persistent dissociation between physical tissue damage and subjective reports. In alignment with the 2020 International Association for the Study of Pain definition, pain is a personal experience filtered through a latent “susceptibility architecture.” While clinical assessment currently relies on static, text-based questionnaires, these are often confounded by linguistic interpretation bias and cognitive literacy. We hypothesized that an individual’s internal psychological substrate—traditionally captured via text—can be characterized through real-time behavioral signatures during physical challenge.

**Objective:**

This study aimed to demonstrate that the “pain-prone” phenotype can be identified through high-frequency digital assessment of pain ratings. By correlating established psychometric traits with dynamic behavioral signatures, we sought to establish a foundation for “digital phenotyping” that moves beyond the limitations of linguistic self-reports.

**Methods:**

A cohort of 534 healthy volunteers (mean age 38.62, SD 22.35 years; n=336, 62.9% male and n=198, 37.1% female) underwent a controlled thermal stimulation protocol (36 °C, 44 °C, 46 °C, and 48 °C). Continuous pain intensity was recorded via a high-frequency (1000 Hz) digital visual analog scale (VAS). To establish a psychological baseline, participants were profiled using the Revised NEO Personality Inventory (NEO PI-R) and the Relationship Questionnaire. Two behavioral indexes were then derived from the digital VAS: the temporal augmentation index (TAI), reflecting within-stimulus physiological sensitization, and the cognitive contrast effect (evaluative instability). Statistical significance was adjusted using the false discovery rate.

**Results:**

Repeated-measure multivariate ANOVA confirmed a highly significant main effect of time for all noxious conditions (*P*<.001; 46 °C: *t*_533_=27.69). Perceived intensity at 46 °C was significantly lower following 48 °C (mean VAS 12.31; SD 15.55) than following 36 °C (mean VAS 30.45; SD 22.38; *t*_533_=−25.76; *P*<.001). Crucially, vulnerability (facet N6 of the NEO PI-R) was significantly associated with contrast magnitude (*q*=.03) and showed a trend for the TAI (*q*=.09), whereas self-discipline (facet C5) showed a significant negative association with the TAI (*q*=.048) and a trend for contrast magnitude (*q*=.09). Mediation analysis identified 2 distinct pathways: (1) a “stabilization path” where secure attachment fully mediated the inhibitory effect of facet C5 on evaluative instability (direct effect *c*′=−0.25; *P*=.11) and (2) an “instability path” where facet N6 exerted a direct amplifying effect on instability (*c*′=0.34; *P*=.03).

**Conclusions:**

Subjective pain evaluation is governed by a stable internal psychological substrate. By shifting the assessment modality from linguistic self-reports to dynamic behavioral signatures, we provide a framework for “digital phenotyping.” These evaluation patterns serve as an objective behavioral marker, enabling the identification of latent susceptibility before chronification and offering a novel foundation for personalized precision pain management.

## Introduction

### The Subjectivity of Pain and the Evolution of the Clinician’s Paradox

The transition from acute to chronic pain remains one of the most formidable frontiers in modern clinical medicine [1]. Traditionally, pain was viewed through a Cartesian, dualistic lens, assuming a linear, proportional relationship between noxious input and perceived intensity. In this classic biomedical model, pain was considered a direct symptom of tissue damage, much like a ringing bell signals a pull on a cord. However, clinical reality has long presented a striking paradox: many patients experience debilitating chronic pain in the absolute absence of identifiable pathology, whereas others recover fully from severe physical trauma without developing long-lasting sensitivity [2-4]. This “clinician’s paradox” suggests that the nervous system is not a passive conduit but an active and plastic interpreter of sensory signals.

In 2020, the International Association for the Study of Pain formalized the shift away from the biomedical model by revising the definition of pain to “an unpleasant sensory and emotional experience associated with, or resembling that associated with, actual or potential tissue damage” [2]. A pivotal aspect of this revision is the explicit recognition that pain is “always a personal experience,” influenced to varying degrees by a complex and dynamic interplay of biological, psychological, and social factors [5,6]. This suggests that pain is an “emergent property” of the brain’s integrative processes rather than a mere sensory readout [7,8]. From this perspective, the initial physical insult serves merely as a “triggering event,” whereas the eventual trajectory toward chronicity is largely governed by the individual’s inherent susceptibility—the “pain-prone” phenotype [9,10].

### Personality and Attachment as Latent Psychological Filters

In accordance with the diathesis-stress model, preexisting psychological architectures function as a latent “vulnerability filter” that determines how raw sensory signals are processed, interpreted, and emotionally modulated [11,12]. Personality traits, particularly those elucidated by the five-factor model, provide a high-resolution map of this temperament. Previous research has consistently demonstrated that individuals with high levels of neuroticism exhibit heightened pain hypersensitivity and a greater predisposition toward maladaptive catastrophizing [4,10,13-15]. Conversely, traits such as conscientiousness and self-discipline appear to foster adaptive coping strategies and higher self-efficacy, potentially acting as a neurocognitive buffer against the amplification of pain signals [16,17].

Furthermore, these traits are deeply intertwined with “internal working models” (IWMs) of attachment. Attachment theory offers a fundamental developmental framework for understanding how early relationship experiences shape a person’s cognitive and emotional templates for regulating distress in response to threat [18-20]. These IWMs act as the brain’s “operating system” for managing physical and psychological pain, serving as a “cognitive calibrator.” Secure attachment provides a stable foundation for the accurate appraisal of threat and the efficient downregulation of distress. In contrast, insecure attachment styles are associated with the hyperactivation of the stress response, causing individuals to exhibit greater fluctuations and evaluative instability in their pain reports [21,22]. We hypothesize that this IWM is the critical nexus through which personality traits are translated into sensory experiences.

### Mechanistic Pathways: Physiological Sensitization vs Cognitive Calibration

To move toward the clinical ideal of “precision pain management,” it is essential to identify the precise mechanisms—both bottom-up and top-down—through which these inherent traits modulate the pain experience. This study focused on 2 distinct behavioral indexes: the temporal augmentation index (TAI) and the cognitive contrast effect. The TAI, conceptually linked to central sensitization, reflects the progressive, time-dependent amplification of nociceptive signaling within the central nervous system during sustained, tonic noxious stimulation [14,23]. This process primarily reflects physiological sensitization at the spinal and supraspinal levels and is a clinical hallmark of the “sensitization-prone” phenotype [23].

Simultaneously, we examined the cognitive contrast effect—a phenomenon where the evaluation of a current noxious stimulus is significantly skewed by the intensity of the preceding sensory context. This effect reflects higher-order cognitive judgments and the stability (or instability) of subjective perception. This process is deeply rooted in the predictive coding framework, which suggests that the brain is an “inference machine” that constantly compares incoming nociceptive input against predicted internal mental models [24,25]. Identifying whether psychological susceptibility primarily modulates “physiological sensitization” (TAI) or “cognitive calibration” (contrast effect) is a critical step for the proactive stratification of patients who are at risk of pain chronification [5,26].

### Beyond Linguistic Modalities: From Text to Behavioral Signatures

Despite the critical role of psychological factors, current clinical assessment relies almost exclusively on traditional, text-based self-report questionnaires. However, these psychometric instruments possess inherent limitations that may significantly compromise the accuracy of patient profiling. The validity of self-reports is frequently confounded by variations in linguistic comprehension, cognitive literacy, and the subjective interpretation of descriptive items [27]. Research in psychometrics has highlighted that individuals may interpret the same written prompt in vastly different ways based on their cognitive load or personal semantics, leading to “common method bias” and a failure to accurately capture the latent psychological construct [28]. It must be acknowledged that visual analog scales (VASs), while providing a continuous metric, are also susceptible to individual interpretational variability similarly to linguistic prompts. However, this study aimed to move beyond treating this variability as “noise” and, instead, treat the dynamic, high-frequency evaluation pattern itself as a characteristic behavioral signature.

To overcome these fundamental limitations, this study proposes a paradigm shift in assessment modality: from linguistic self-reports to dynamic behavioral evaluation. By analyzing the real-time “behavioral signature” of pain ratings during controlled physical stimulation, we aimed to bridge the gap between abstract psychological constructs and observable neurobehavioral reactions. We hypothesized that stable personality facets—previously captured only through text—can be characterized more accurately through the unique ways in which an individual’s pain ratings fluctuate and augment in response to noxious input. This transition from “what the patient says” to “how the patient reacts to a stimulus” allows for a multimodal understanding of human nature, redefining psychological traits not just as abstract descriptions but as specific patterns of behavioral reaction. This approach provides a robust foundation for “digital phenotyping” in pain medicine [29-31]. The primary aim of this study was to demonstrate that dynamic behavioral signatures of pain evaluation reflect an individual’s latent psychological substrates. We hypothesized that (1) personality traits such as vulnerability (facet N6 of the Revised NEO Personality Inventory [NEO PI-R]) would amplify both physiological sensitization (the TAI) and cognitive evaluative instability (the contrast effect) and (2) this instability can be explained through 2 distinct psychological pathways—a “stabilization path” and an “instability path”—mediated by IWMs of attachment.

## Methods

### Ethical Considerations

This study was approved by the Research Ethics Committee of the Graduate School of Frontier Biosciences, Osaka University (approval FBS2020-13 and FBS2021-10), and was conducted in strict accordance with the Declaration of Helsinki. All participants provided written informed consent before participation. To ensure participant privacy and confidentiality, all data were pseudonymized (symbolized) using identification numbers, and the correspondence table between personal information and these numbers was strictly managed by the research team. Data processed on external cloud servers contained no personally identifiable information. Participants were compensated for their time with a payment of JP ¥1000 (US $7.28) per hour.

### Recruitment and Procedures

Participants were recruited through 2 primary channels to ensure a diverse age distribution: (1) KOAN, an internal online bulletin board for students and staff at Osaka University; and (2) the Toyonaka City Silver Human Resources Center targeting community-dwelling older adults. The recruitment advertisements specified that participants must be aged at least 18 years and in good physical and mental health.

To ensure participant safety and data validity, we applied strict exclusion criteria. Individuals were ineligible if they had (1) known neurological disorders or a history of brain-related conditions (eg, epilepsy, stroke, or organic brain disease), (2) a clinical diagnosis of chronic pain or current use of narcotic analgesics, (3) electronic implants such as cardiac pacemakers, or (4) severe visual impairment that would interfere with using the digital tablet for pain assessment. Additionally, candidates were informed that thermal stimulation might cause temporary skin redness; those who did not consent to this potential effect were excluded.

The systematic screening followed a 3-step process. First, applicants contacted the research team via email and completed a health screening questionnaire based on the above criteria. Second, a researcher verified their eligibility. Third, for eligible candidates, a research assistant coordinated the experimental sessions via email. All experiments were conducted in a dedicated laboratory at Osaka University (Japan Registry of Clinical Trials jRCT1090220286).

A total of 538 healthy volunteers were initially recruited through these systematic procedures. Of the initial cohort of 538 participants, 4 (0.7%) were excluded due to a significant discrepancy between their real-time VAS ratings and postexperimental verbal reports (numeric rating scale), suggesting equipment misuse or measurement error. The final analytical sample consisted of 534 participants (mean age 38.62, SD 22.35 years; n=336, 62.9% male and n=198, 37.1% female). For multivariate analyses involving psychological inventories, the effective sample size ranged from 522 to 530 due to minor missing data.

### Psychological Profiling

Before the thermal stimulation trials, participants’ psychological architectures were profiled using 2 validated instruments. The first, the NEO PI-R (Japanese version), is a 240-item questionnaire assesses the “big five” domains (neuroticism, extraversion, openness, agreeableness, and conscientiousness) and their 30 constituent facets. The Japanese version has demonstrated high reliability and validity across diverse demographic groups [21,27]. The second was the Relationship Questionnaire, an instrument that measures adult attachment styles—secure, dismissing, preoccupied, and fearful—based on mental models of the self and others. Participants provide self-ratings on a 7-point Likert scale for each of the 4 prototypes, allowing for both categorical and dimensional analyses of their IWMs [22,28,32].

### Thermal Stimulation Protocol

Thermal stimuli were delivered via a computer-controlled PATHWAY system (Medoc - Advanced Medical Systems Ltd) using a contact advanced thermal stimulator probe (30 × 30 mm) attached to the medial aspect of the left forearm. The Medoc PATHWAY system is a gold-standard device in quantitative sensory testing (QST) research capable of precise temperature delivery with a 0.1 °C resolution.

The experimental program consisted of 20 tonic heat stimuli, each lasting 30 seconds at a target temperature, with an interstimulus interval of 30 seconds at a baseline of 35 °C to avoid excessive cumulative skin heating. The target temperatures included 1 nonnoxious control (36 °C) and 3 noxious conditions (44 °C, 46 °C, and 48 °C). The stimuli were presented in a fixed pseudorandomized sequence (36, 44, 48, 46, 46, 44, 36, 48, 46, 48, 36, 44, 48, 44, 36, 46, 36, 44, 48, and 46) to control for order effects while specifically enabling the assessment of temperature-dependent contrast effects on the 46 °C target. Each stimulus ramped from baseline to the target temperature at a rate of 1 °C per second and returned to baseline at the same rate. The heating ramp of 1 °C per second preferentially activates unmyelinated C-fibers, which are known to contribute to slow, burning pain and temporal summation phenomena.

### Real-Time Pain Assessment

Participants were instructed to rate their pain intensity continuously throughout the stimulation period using a sliding-lever VAS device (Intercross-210; Intercross Corporation). The VAS was anchored at 0 (“no pain”) and 100 (“maximum imaginable pain”). The digital output was sampled at a high frequency (1000 Hz) to ensure high-fidelity data acquisition and facilitate effective noise reduction during subsequent signal processing.

### Derivation of Modulation Indexes

Two distinct modulation indexes were calculated for each participant.

First, the TAI: the 30-second stimulation period was divided into three 10-second epochs (time A: 0-10 seconds; time B: 10-20 seconds; time C: 20-30 seconds). After preliminary examination of all possible increments between the intervals (B – A, C – B, and C – A), the C – A index (the difference in mean VAS between the terminal and initial phases) was adopted as the primary measure of within-stimulus pain augmentation as it most clearly and robustly reflected the overall progression of pain intensity. It should be noted that, while the ratio-based windup index (VAS[C]/VAS[A]) is frequently used in clinical populations, it was not used in our primary analyses. Because our cohort consisted of healthy volunteers, initial pain ratings (time A) were often near 0, creating mathematical instability and artificially inflated ratios. Thus, the absolute difference (C – A) was adopted for mathematical robustness.

Second, the cognitive contrast effect: this index quantified the influence of preceding thermal context on the evaluation of the 46 °C target stimulus. The “magnitude of the contrast effect” was defined as the difference in VAS scores between the low-contrast condition (preceded by 36 °C) and the high-contrast condition (preceded by 48 °C).

### Statistical Analysis

All analyses were conducted using JMP (version 14.2.0; JMP Statistical Discovery LLC). Data distribution was assessed using the Shapiro-Wilk test, which revealed significant nonnormality for most behavioral indexes (*P*<.05). Consequently, the Spearman rank correlation coefficient (ρ) was used to evaluate associations between psychological facets and pain modulation indexes. To control for the inflation of type I error rate due to multiple comparisons across personality facets and attachment prototypes, the false discovery rate (FDR) using the Benjamini-Hochberg procedure was applied, with *q* values (FDR-adjusted *P* values) of <.05 considered statistically significant. Repeated-measure multivariate ANOVA was configured as a 4 (temperature: 36 °C, 44 °C, 46 °C, and 48 °C) × 3 (time: epoch A, B, and C) factorial design, where 36 °C served as the non-noxious control condition. Greenhouse-Geisser corrections were applied to the df across all temperature levels to adjust for violations of sphericity when evaluating time-dependent changes in VAS scores across these temperature conditions. Evaluation instability was operationally defined as the magnitude of the cognitive contrast effect. Additionally, participants were categorized into low- and high-sensitivity groups via a median split based on their average VAS score at 46 °C to investigate group-level differences in temporal augmentation. Finally, a mediation analysis was performed following the standard criteria of Baron and Kenny [33]. The indirect effects were further validated using the Sobel test to ensure statistical robustness. For all analyses not involving multiple comparisons (eg, multivariate ANOVA and mediation), significance was defined as *P*<.05. All *t* tests were 2-tailed.

## Results

### Time-Dependent Modulation of Pain

**Figure 1:**
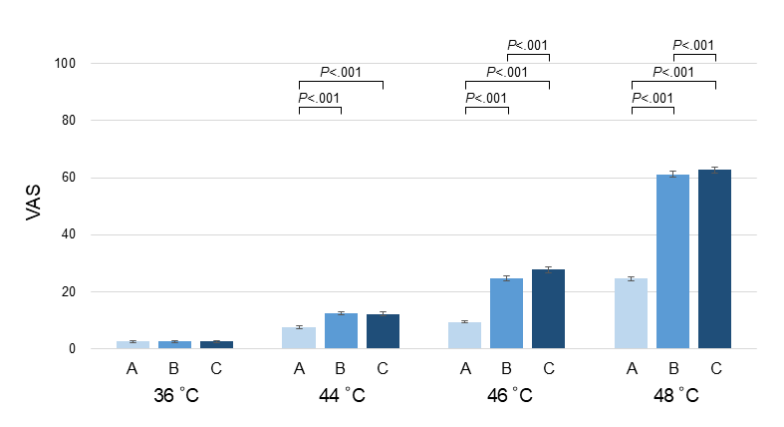
Time course of pain intensity ratings within the 30-second heat stimulation period. The 30-second period is divided into 10-second intervals (epochs A, B, and C). The temporal augmentation index is calculated as C – A. Significant main effects of time were observed for all noxious conditions (*P*<.001). Data are presented as mean and SE (n=534).

The model confirmed a highly significant main effect of time for all noxious conditions (44 °C, 46 °C, and 48 °C; *P*<.001 in all cases). For the 44 °C condition, the mean VAS increased from 7.52 (SD 9.76) at time A to 12.20 (SE 0.62) at time C (*P*<.001). For the 46 °C target stimulus, univariate results revealed a linear progression of pain intensity over the 30-second window. Significant increases were observed between time A and B (t_533_=27.69; *P*<.001), time A and C (t_533_=26.88; *P*<.001), and time B and C (t_533_=12.33; *P*<.001). For the 48 °C condition, a more pronounced augmentation was observed, rising from 24.62 (SE 0.68) at time A to 62.73 (SE 1.07) at time C (*P*<.001). Overall, these results demonstrate a robust and sustained temporal augmentation (TAI; Figure 1). In contrast, the 36 °C control condition showed no significant variation across the 3 epochs even within this factorial framework (*F*_1.31, 695.56_=2.43; *P*=.11). This confirms that the observed augmentation reflects specific nociceptive temporal integration rather than generalized response fatigue or habituation.

### Context-Dependent Modulation (Contrast Effect)

A paired *t* test revealed a powerful contrast effect on the 46 °C target stimulus (t_533_=−25.76; *P*<.001). Perceived intensity for the 46 °C stimulus was significantly lower in the high-contrast condition (following 48 °C; mean VAS 12.31, SD 15.55) than in the low-contrast condition (following 36 °C; mean VAS 30.45, SD 22.38). This confirms that subjective pain evaluation is not an absolute readout of temperature but a relative judgment influenced by immediate sensory history and the brain’s contextual predictive model.

### Correlation Analysis With Personality and Attachment

**Figure 2:**
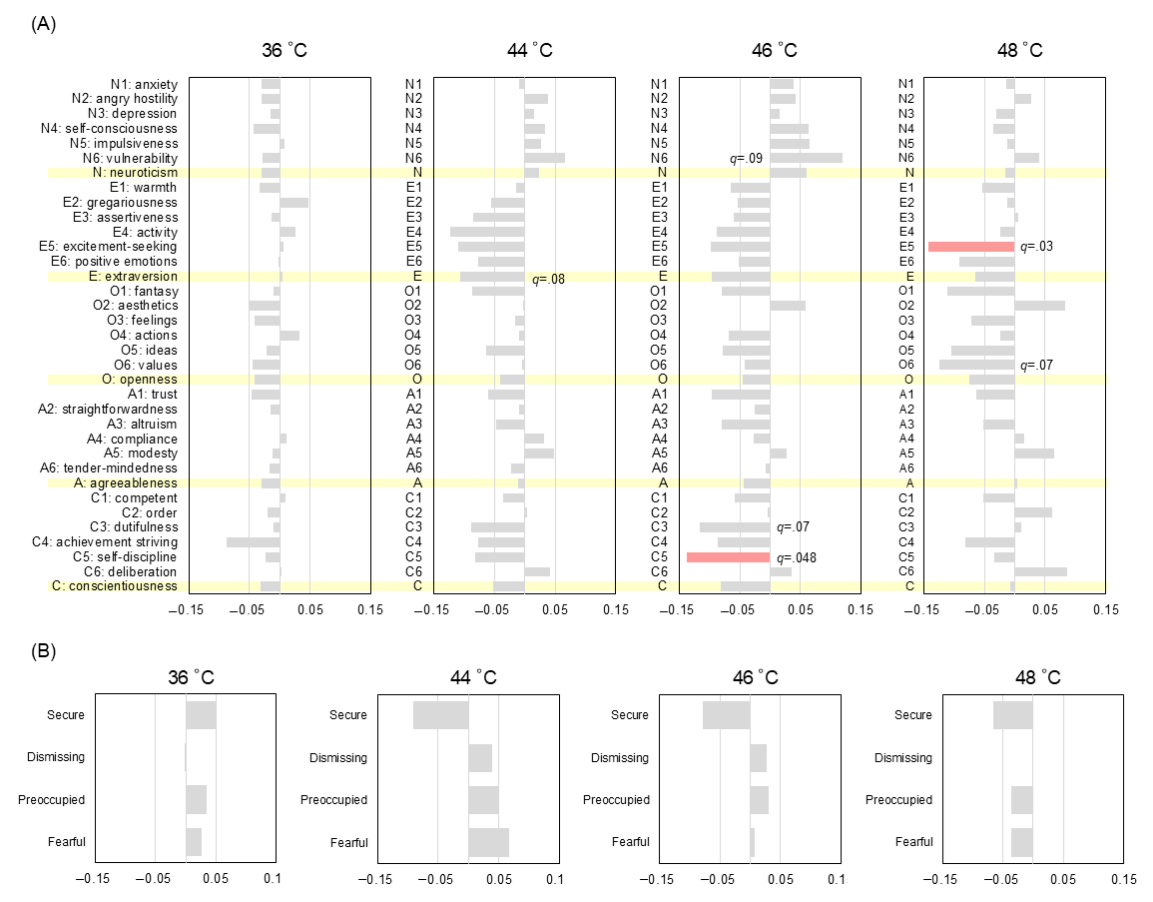
Correlations among personality traits, attachment styles, and intrastimulus changes in pain ratings (C – A) across stimulus temperatures. (A) Spearman rank correlation coefficients (ρ) between the “big five” personality facets and the temporal augmentation index (TAI; defined as epoch C – epoch A). Rightward and leftward bars indicate positive and negative correlations, respectively; shaded rows highlight the 5 main domains. Significance was adjusted using the Benjamini-Hochberg false discovery rate procedure (n=523). (B) Correlations between attachment styles and the TAI (n=530).

**Figure 3:**
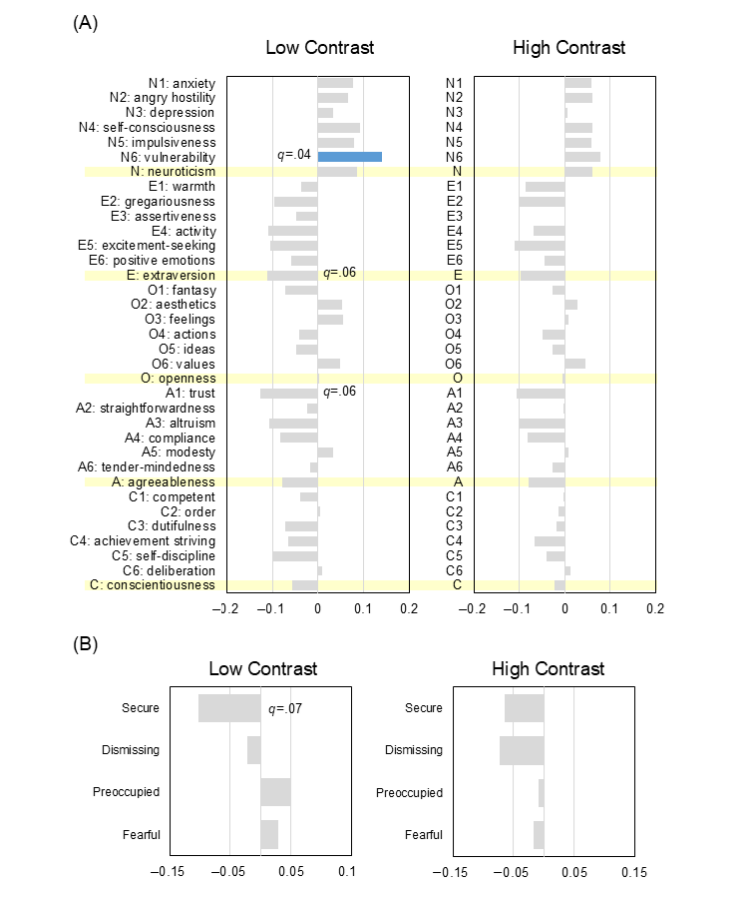
Correlations among personality traits, attachment styles, and average pain intensity at 46 °C in low- and high-contrast conditions. (A) Spearman rank correlation coefficients (ρ) between the “big five” personality facets and average pain intensity in the low-and high-contrast condition. Significance was adjusted using the Benjamini-Hochberg false discovery rate procedure (n=523). (B) Correlations between attachment styles and average pain intensity in the low-and high-contrast condition (n=530).

**Figure 4:**
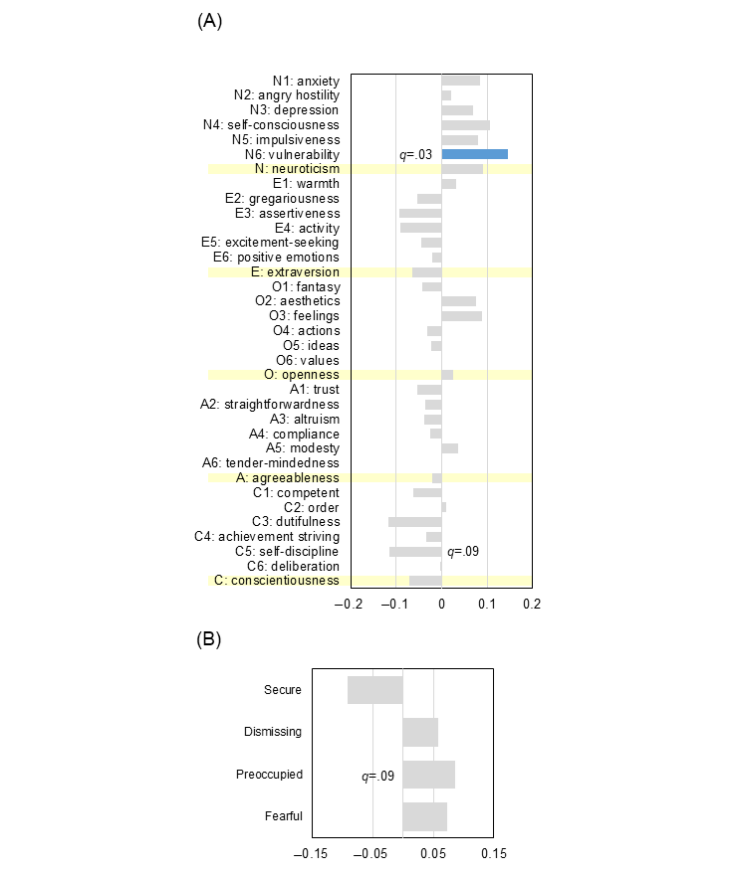
Correlations among personality traits, attachment styles, and the magnitude of the contrast effect at 46 °C. (A) Spearman rank correlation coefficients (ρ) between the “big five” personality facets and the magnitude of the contrast effect (defined as the difference in average visual analog scale scores between low- and high-contrast conditions). Significance was adjusted using the Benjamini-Hochberg false discovery rate procedure (n=523). (B) Correlations between attachment styles and the contrast effect (n=530).

Correlation analysis using the Spearman ρ and the FDR following the Benjamini-Hochberg procedure revealed that self-discipline (C5) was significantly negatively associated with the TAI at 46 °C (Figure 2A; ρ=−0.14; *q*=.048). Vulnerability (facet N6 of the NEO PI-R) and dutifulness (facet C3) were observed as trends within the FDR-adjusted framework (N6: ρ=0.12 and *q*=.09; C3: ρ=−0.12 and *q*=.07). The broad domain of neuroticism was not significantly associated with the 46 °C TAI (ρ=0.06; *q*=.27). In contrast, N6 showed significant positive correlations with average pain intensity in the low-contrast condition (Figure 3A; ρ=0.14; *q*=.04) and with the magnitude of the contrast effect (Figure 4A; ρ=0.15; *q*=.03). Notably, the associations for N6 with these indexes remained significant after FDR correction, whereas the broad domain of neuroticism was not significantly associated with these indexes after correction (low contrast: ρ=0.09 and *q*=.12; contrast effect: ρ=0.09 and *q*=.18). However, the association between C5 and the contrast effect was observed as a trend (ρ=−0.11; *q*=.09). Additionally, at the highest temperature of 48 °C, excitement seeking (facet E5) exhibited a significant negative association with TAI after FDR correction (ρ=−0.14; *q*=.03).

**Figure 5:**
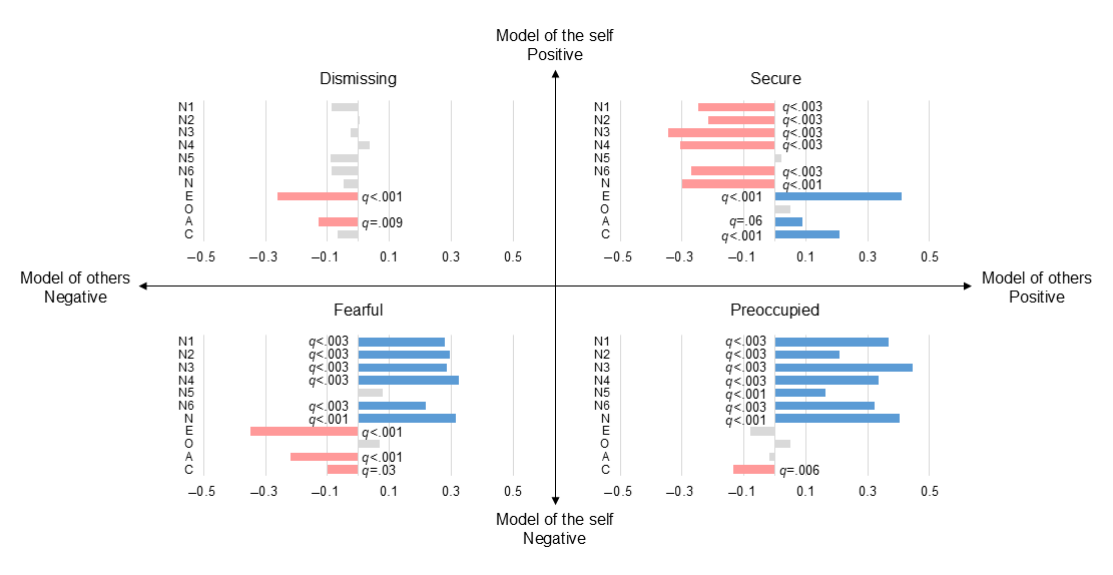
Correlations between attachment styles and personality traits based on the mental models of the self and others. The matrix displays Spearman rank correlation coefficients (ρ) between the 4 attachment prototypes and the “big five” personality domains. The prototypes are organized according to the internal working models of the self (horizontal axis) and others (vertical axis). Significance was adjusted using the Benjamini-Hochberg false discovery rate procedure (n=522).

Regarding attachment styles, no prototypes were directly correlated with the TAI (*q*>.05 in all cases; Figure 2B), suggesting that temporal augmentation may be governed by more rudimentary neurobiological sensitization paths. However, IWMs were significantly linked to evaluative stability. The secure attachment style showed a negative correlation with pain intensity in the low-contrast condition, appearing as a trend (Figure 3B; ρ=−0.10; *q*=.07). Furthermore, the magnitude of the contrast effect was associated with the preoccupied prototype as a trend (Figure 4B; ρ=0.09; *q*=.09), whereas the association with the secure prototype did not maintain significance within the FDR framework (ρ=−0.09; *q*=.13). These relationships were further supported by significant correlations between attachment styles and personality domains (Figure 5; with many associations reaching *q*<.001); for instance, the secure style was negatively associated with neuroticism (ρ=−0.30; *q*<.001) and positively associated with extraversion (ρ=0.41; *q*<.001), whereas the preoccupied style was linked to high neuroticism (ρ=0.40; *q*<.001).

### Mediation Analysis: Stabilization vs Instability

**Figure 6:**
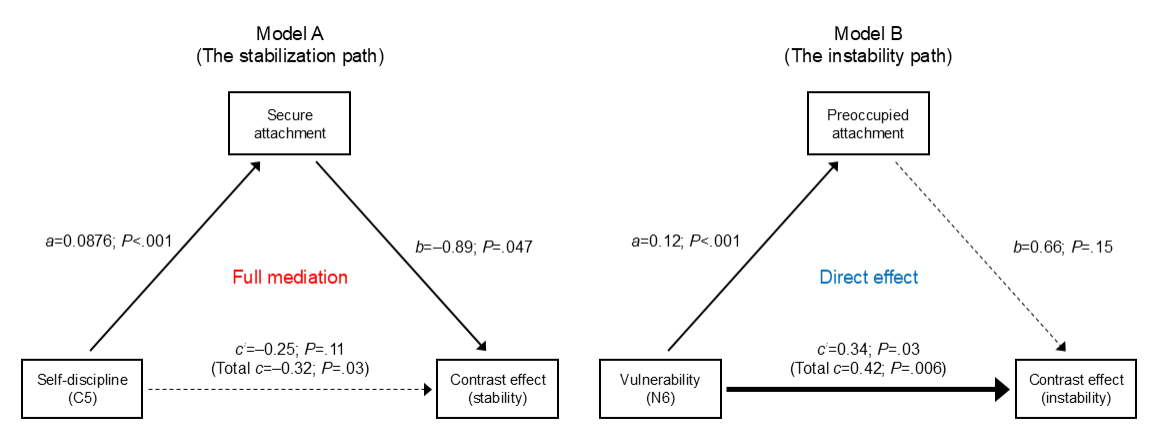
Path models of the effects of personality traits and attachment styles on the magnitude of the contrast effect. The path diagrams illustrate 2 distinct psychological mechanisms governing the stability of pain evaluation (model A: stabilization path; model B: instability path). Numbers on the paths represent unstandardized coefficients (B), with values in parentheses indicating the total effect. Solid and dashed arrows denote significant and nonsignificant paths, respectively. Highlighted labels denote significant negative (suppression) and positive (amplification) impacts on the contrast effect. Significant paths were defined at *P*<.05 (n=522).

The mediation findings provided the deepest insight into the psychological architecture of pain evaluation (Figure 6):

Model A, the stabilization path: the inhibitory effect of self-discipline (facet C5) on evaluation instability was fully mediated by secure attachment. The direct effect of C5 became nonsignificant (c′=−0.25; *P*=.11) when the mediator was included. This implies that discipline promotes stability primarily by fostering a secure internal mental framework.

Model B, the instability path: the amplifying effect of vulnerability (N6) on evaluation instability remained a direct effect (c′=0.34; *P*=.03). Although N6 strongly predicted preoccupied attachment, the attachment style itself did not significantly influence the contrast effect in this specific model, suggesting a direct temperamental “destabilization” in vulnerable individuals.

### Impact of Baseline Pain Sensitivity on Temporal Augmentation and Contrast Effect

**Figure 7:**
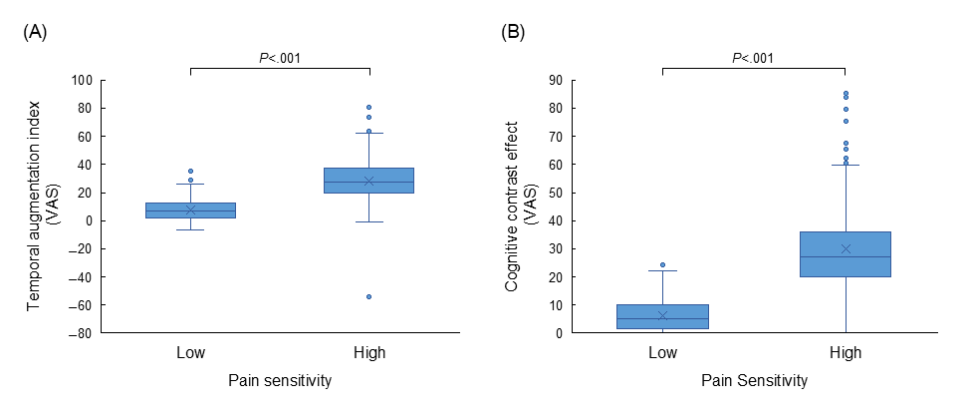
Comparison of pain modulation indexes between groups with different baseline pain sensitivity. Participants were stratified into low- and high-sensitivity groups based on a median split of average visual analog scale (VAS) scores at 46 °C. The panels represent (A) the temporal augmentation index and (B) the cognitive contrast effect. Box plots indicate the median, IQR, and range, with individual data points overlaid. Significance was determined using the Wilcoxon rank sum test (N=534).

To further elucidate the clinical relevance of our findings, participants were stratified into low– and high–pain sensitivity groups based on a median split of their average VAS scores at 46 °C. Nonparametric comparisons using the Wilcoxon rank sum test revealed that the high-sensitivity group exhibited a significantly larger TAI (mean 28.52, SD 15.10) than the low-sensitivity group (mean 7.92, SD 7.11; *z*=16.25; *P*<.001). Furthermore, the cognitive contrast effect was also significantly greater in the high-sensitivity group (mean 30.13, SD 14.70) than in the low-sensitivity group (mean 6.15, SD 5.13; *z*=19.09; *P*<.001). These results indicate that individuals with inherently higher pain sensitivity are more susceptible to both temporal sensitization (windup) and context-dependent evaluation instability (Figure 7).

## Discussion

### Principal Findings

In this cross-sectional study of 534 healthy adults, we demonstrated that specific personality facets were selectively associated with dynamic behavioral signatures of pain modulation under standardized tonic heat stimulation. Vulnerability (facet N6 of the NEO PI-R) was associated with greater evaluative instability, whereas self-discipline (facet C5) was associated with reduced temporal augmentation. Importantly, these associations were observed at the facet level rather than uniformly across broader personality domains, suggesting selective mechanistic relationships rather than a generalized negative affect effect [4,10,13,14].

### The Subjectivity of Pain: The Observer as the Primary Architect

The findings of this study provide empirical evidence that the “observer” plays a critical role in shaping the pain experience, extending beyond a purely Cartesian view of nociception [2]. By standardizing the physical input (46 °C) while observing substantial interindividual variation in both the TAI and the cognitive contrast effect, we demonstrated that pain is not merely a passive sensory readout but reflects integrative brain processes [5,6,16,34].

Specifically, the TAI used in this study is conceptually linked to the clinical phenomenon of temporal summation [23], which has been described as a behavioral correlate of central sensitization [14,23]. Temporal summation reflects activity-dependent amplification of nociceptive signaling within the central nervous system and has been extensively characterized in experimental and clinical research [14,23]. The positive correlation between vulnerability (N6) and TAI suggests that this trait may be associated with increased sensitivity to sustained nociceptive input.

However, although the TAI was conceptually linked to central sensitization, we did not directly measure neurophysiological markers such as conditioned pain modulation or spinal nociceptive reflexes [35,36]. Therefore, our behavioral indexes should be interpreted as indirect proxies rather than definitive measures of central sensitization.

Pain, as an emergent property of consciousness, is not solely a reflection of peripheral input but is shaped by latent psychological architecture. Even under identical physical conditions, the perceived intensity and its temporal dynamics varied substantially across individuals.

### Inherent Susceptibility: Vulnerability (N6) and Sensitization Processes

A central pillar of our findings is the susceptibility hypothesis, which proposes that individual differences in psychological architecture are associated with variability in nociceptive modulation. Vulnerability reflects a temperamental disposition characterized by emotional reactivity and perceived difficulty coping with stress. Individuals with higher N6 scores exhibited greater temporal augmentation and evaluative instability during sustained stimulation.

While this pattern is conceptually consistent with models of central sensitization [14,23], this study cannot establish causal or longitudinal risk relationships. Therefore, the observed associations should be interpreted as correlational markers of susceptibility rather than determinants of future chronification [9,26,37].

### The Stabilization Path: Attachment as a Cognitive Calibrator

We identified a stabilization pathway in which secure attachment mediates the relationship between self-discipline (C5) and evaluative stability.

According to attachment theory, IWMs regulate threat appraisal and distress tolerance [18-20,38]. Secure attachment has been associated with adaptive emotion regulation and reduced pain-related distress [21,22]. Our mediation findings extend this literature by suggesting that attachment-related internal models may influence not only pain intensity but also the stability of pain evaluation across contextual changes.

### Mechanistic Implications: Predictive Coding and Evaluative Noise

The contrast effect observed in our results aligns with predictive coding frameworks in which perception reflects the integration of sensory input and prior expectations [24,25]. Therefore, pain is evaluated relative to contextual history rather than as an absolute signal.

Individuals high in vulnerability (N6) exhibited larger contrast effects, suggesting increased contextual sensitivity. Within predictive processing models, this may reflect heightened responsiveness to prediction error [39]. These findings support the view that psychological architecture influences the stability of evaluative processes during nociceptive challenge [40].

### Modality Shift: From Text-Based Introspection to Behavioral Signatures

This study proposes a complementary assessment modality that integrates dynamic behavioral evaluation with established psychometric measures [29,30].

QST has long been a cornerstone of pain phenotyping [23,41,42]. Rather than replacing QST, our approach links dynamic QST-derived indexes to latent psychological traits within a large cohort.

By focusing on within-subject temporal change (C – A) and contextual contrast, we aimed to minimize reliance on absolute VAS magnitude, which is known to vary across individuals due to interpretational and response-style differences [27,28]. In this sense, dynamic evaluation patterns may serve as behavioral expressions of psychological structure.

### Limitations

Several limitations should be acknowledged. First, while the five-factor model is widely used, it does not fully capture the dynamic and context-sensitive nature of personality. Alternative dimensional models (eg, the HEXACO model [43] or hierarchical models of affect regulation) may offer complementary perspectives. Second, although temporal augmentation was conceptually linked to central sensitization [14,23], we did not directly measure neurophysiological markers (eg, conditioned pain modulation [35,42,44,45] or spinal reflexes). Therefore, our behavioral indexes should be interpreted as proxies rather than definitive measures of central sensitization. Third, because the heating rate was 1 °C per second, the stimulation predominantly engaged C-fiber–mediated nociception [14,23]. Therefore, the findings may not generalize to A-delta fiber–mediated sharp pain paradigms. Fourth, as this was a cross-sectional study of healthy volunteers, longitudinal prediction of chronic pain development cannot be inferred [9,26,37]. Finally, although statistically significant, the observed effect sizes were modest, reflecting the multifactorial nature of pain modulation [5,6].

### Conclusions

In conclusion, the findings suggest that dynamic pain evaluation patterns are associated with stable psychological substrates. By integrating personality facets, attachment models, and behavioral indexes derived from standardized experimental stimulation, this study contributes to the development of multimodal pain phenotyping frameworks [17,46,47].

Future longitudinal studies will be necessary to determine whether these behavioral signatures have predictive value for pain chronification [9,44]. These results provide a foundation for further investigation into how psychological architecture relates to dynamic nociceptive processing.

## Appendix

Multimedia Appendix 1 STROBE checklist.

## Abbreviations

FDR: false discovery rate
IWM: internal working model
NEO PI-R: Revised NEO Personality Inventory
QST: quantitative sensory testing
TAI: temporal augmentation index
VAS: visual analog scale

## Data Availability

The datasets generated or analyzed during this study are not publicly available to ensure participant confidentiality and comply with ethical restrictions. However, they may be available from the corresponding author on reasonable request.
