# Supplementary material for: Digital Phenotyping of Pain Modulation and Associations Among Personality, Attachment, and Behavioral Signatures: Cross-Sectional Study
Source: JMIR Form Res. 2026 Mar 13;10:e91540. doi: 10.2196/91540 (PMC13032095; doi:10.2196/91540)
Supplement: Multimedia Appendix 1 [file formative_v10i1e91540_app1.docx]

# Supplementary File: STROBE Checklist (Verbatim Version)

**Manuscript Title:** Digital Phenotyping of Pain Modulation—Associations Between Personality, Attachment, and Behavioral Signatures: Cross-Sectional Study

**Study Design:** Cross-sectional observational study

The checklist below follows the official STROBE Statement (Strengthening the Reporting of Observational Studies in Epidemiology) for cross-sectional studies. The rightmost column indicates where each item is addressed in the revised manuscript.

| Item No. | Section | STROBE Recommendation (Verbatim) | Location in Manuscript |
| --- | --- | --- | --- |
| 1 | Title and abstract | Indicate the study’s design with a commonly used term in the title or the abstract; Provide in the abstract an informative and balanced summary of what was done and what was found. | Title; Abstract |
| 2 | Background/rationale | Explain the scientific background and rationale for the investigation being reported. | Introduction |
| 3 | Objectives | State specific objectives, including any prespecified hypotheses. | End of Introduction |
| 4 | Study design | Present key elements of study design early in the paper. | Methods – Recruitment and Procedures |
| 5 | Setting | Describe the setting, locations, and relevant dates, including periods of recruitment, exposure, follow-up, and data collection. | Methods – Recruitment and Procedures |
| 6 | Participants | Give the eligibility criteria, and the sources and methods of selection of participants. | Methods – Recruitment and Procedures |
| 7 | Variables | Clearly define all outcomes, exposures, predictors, potential confounders, and effect modifiers. Give diagnostic criteria, if applicable. | Methods – Derivation of Modulation Indices |
| 8 | Data sources/measurement | For each variable of interest, give sources of data and details of methods of assessment (measurement). Describe comparability of assessment methods if there is more than one group. | Methods – Psychological Profiling; Thermal Protocol |
| 9 | Bias | Describe any efforts to address potential sources of bias. | Methods – Statistical Analysis; Discussion – Limitations |
| 10 | Study size | Explain how the study size was arrived at. | Methods – Recruitment (final sample description) |
| 11 | Quantitative variables | Explain how quantitative variables were handled in the analyses. If applicable, describe which groupings were chosen and why. | Methods – Statistical Analysis |
| 12 | Statistical methods | Describe all statistical methods, including those used to control for confounding; Describe any methods used to examine subgroups and interactions; Explain how missing data were addressed; Describe any sensitivity analyses. | Methods – Statistical Analysis |
| 13 | Participants (Results) | Report numbers of individuals at each stage of study—eg, numbers potentially eligible, examined for eligibility, confirmed eligible, included in the study, completing follow-up, and analyzed. | Results – Sample description |
| 14 | Descriptive data | Give characteristics of study participants (eg, demographic, clinical, social) and information on exposures and potential confounders. | Results – Participant characteristics; Figures |
| 15 | Outcome data | Report numbers of outcome events or summary measures. | Results – TAI and Contrast Effect |
| 16 | Main results | Give unadjusted estimates and, if applicable, confounder-adjusted estimates and their precision (eg, 95% confidence interval). Report category boundaries when continuous variables were categorized. | Results – Correlation and Mediation Analyses |
| 17 | Other analyses | Report other analyses done—eg, analyses of subgroups and interactions, and sensitivity analyses. | Results – Baseline Sensitivity Stratification |
| 18 | Key results | Summarize key results with reference to study objectives. | Discussion – Principal Findings |
| 19 | Limitations | Discuss limitations of the study, taking into account sources of potential bias or imprecision. | Discussion – Limitations |
| 20 | Interpretation | Give a cautious overall interpretation of results considering objectives, limitations, multiplicity of analyses, results from similar studies, and other relevant evidence. | Discussion – Interpretation sections |
| 21 | Generalisability | Discuss the generalisability (external validity) of the study results. | Discussion – Limitations |
| 22 | Funding | Give the source of funding and the role of the funders for the present study and, if applicable, for the original study on which the present article is based. | Funding section |
